# Relationships among peri-traumatic circulating endocannabinoids and long-term, negative outcomes following traumatic injury

**DOI:** 10.1007/s00213-025-06837-4

**Published:** 2025-07-11

**Authors:** Emily A. Albertina, Carissa W. Tomas, Timothy J. Geier, Sydney C. Timmer-Murillo, Isela G. Pina, Kelley Jazinski-Chambers, Garrett Sauber, Jacklynn M. Fitzgerald, Christine L. Larson, Terri A. deRoon-Cassini, Cecilia J. Hillard

**Affiliations:** 1 Department of Psychology, University of Wisconsin, Milwaukee, USA; 2 Medical College of Wisconsin Institute for Health and Equity, Division of Epidemiology and Social Sciences, Milwaukee, USA; 3 Medical College of Wisconsin Comprehensive Injury Center, Division of Data Surveillance & Informatics, Milwaukee, USA; 4 Division of Trauma and Acute Care Surgery, Medical College of Wisconsin, Milwaukee, USA; 5 Department of Pharmacology and Toxicology, Medical College of Wisconsin, Milwaukee, USA; 6 Neuroscience Research Center, Medical College of Wisconsin, Milwaukee, USA; 7 Department of Psychology, Marquette University, Milwaukee, USA

**Keywords:** 2-Arachidonoylglycerol, Anandamide, CAPS-5, PCL-5, PTSD, Chronic pain, Functional engagement, Racially/ethnically marginalized

## Abstract

**Rationale:**

Traumatically injured individuals can develop chronic negative psychological sequelae. Improved understanding of contributing, peri-traumatic risk factors is essential to reduce the risk of these consequences. Previous studies have found that peri-traumatic, circulating endocannabinoid concentrations are positively associated with development of post-traumatic stress disorder (PTSD), chronic pain and depression months later, particularly in members of racial/ethnic groups that have been historically marginalized.

**Objectives:**

This replication study examined relationships among peri-trauma serum endocannabinoid concentrations and long-term consequences in a cohort comprised primarily of individuals from marginalized racial and ethnic groups.

**Methods:**

Participants (*n* = 100; 81% from marginalized racial and ethnic groups) were traumatically injured adults presenting to the ED of an urban tertiary care hospital. Endocannabinoids *N*-arachidonoylethanolamine (AEA) and 2-arachidonoylglycerol (2-AG) were measured in serum collected within days (peri-trauma) and 6–10 months following injury (followup). Assessments, including PTSD, depression, pain and quality of life were completed. Statistical approaches, including multivariate, hierarchical regressions, were used to determine associations among serum endocannabinoid concentrations and long-term outcomes.

**Results:**

Although it did not survive correction for multiple comparisons, peri-trauma serum 2-AG concentrations. Peritrauma serum 2-AG concentrations were also positively associated with PTSD, pain severity, and functional engagement scores at follow-up. There were no significant associations between circulating 2-AG or AEA and depression.

**Conclusions:**

These findings generally replicate earlier studies demonstrating that serum 2-AG concentrations are biomarkers of risk for PTSD and pain and uncover an additional association with poor functional quality of life. Further studies are needed to determine the underlying mechanisms of these relationships.

## Introduction

Traumatic injury is a leading cause of death in the United States, and injury survivors are at greater risk for longterm, adverse physical and emotional outcomes when ompared to the general population (DiMaggio et al. 2016). For example, those experiencing traumatic injury are at greater risk than the population at large to develop post-traumatic stress disorder (PTSD; Alarcon et al. 2012) and PTSD after injury is associated with reduced emotional and functional quality of life (deRoon-Cassini et al. 2010). Similarly, chronic pain is a debilitating condition that occurs in 15% percent of individuals following a traumatic injury (Daoust et al. 2018). Chronic pain (Fine 2011; Speed et al. 2018) and PTSD (Undavalli et al. 2014) have extremely large societal impacts, including loss of work, health care costs and attendant issues of chronic substance use. Traumatic injury is also associated with long-term depression and anxiety (Wiseman et al. 2015). PTSD, chronic pain, and depression are not inevitable consequences of a traumatic injury, and better understanding of the biological effects of trauma could provide important information about risk factors for their development and inform therapies to reduce their incidence.

Preclinical and evolving clinical data support the hypothesis that the endocannabinoid (eCB) system contributes to the risk for the development of long-term, negative outcomes following stress and trauma (deRoon-Cassini et al. 2020; Fitzgerald et al. 2021; Trevino et al. 2022). The eCB system is a neuromodulatory system composed of G-protein-coupled receptors (CB1R and CB2R), endogenous ligands, and metabolic enzymes. CB1Rs are highly expressed in the brain and two eCBs, *N*-arachidonoylethanolamine (AEA) and 2-arachidonoylglycerol (2-AG), activate CB1R-mediated signaling (Hillard 2015). The central nervous system (CNS) eCB system is a critical regulator of synaptic activity and, among other functions, serves as a mechanistic link between stress exposure and changes in synaptic activity (Morena et al. 2016). Rodent models of chronic stress demonstrate alterations in CB1R signaling in brain regions sensitive to stress, including the hippocampus and prefrontal cortex (Hill et al. 2005; Wang et al. 2010; Berger et al. 2018). Behavioral studies in preclinical models demonstrate enhanced eCB-CB1R signaling is associated with reduced anxiety and enhanced extinction of fear memories (Gunduz-Cinar et al. 2023) and elevation of mood (Bedse et al. 2018). While many studies suggest eCB/CB1R signaling serves as a stress buffer and its activation could promote stress adaptation and thereby mitigate stress-related disorders (Bedse et al. 2020), some consequences of stress-induced eCB/CB1R signaling, including generalization of threat memories (Lesuis et al. 2024), enhanced memory consolidation (Campolongo et al. 2009), and reinstatement of drug-seeking behaviors (McReynolds et al. 2018), likely contribute to, rather than mitigate, the long-term consequences of trauma.

The eCBs are also present in the peripheral circulation and multiple studies have examined associations of their concentrations with early life trauma (Marco et al. 2014; Bassir Nia et al. 2019), acute stress responses, exercise, and psychopathology (Hillard 2018). A recent meta-analysis of available studies found that circulating concentrations of both AEA and 2-AG were higher when individuals with any diagnosis (i.e. anxiety, depression and/or PTSD) were compared to those without (Gowatch et al. 2024). However, the only significant relationship when the diagnoses were considered separately was between circulating AEA and anxiety disorders. These findings highlight a need for further work exploring the relationships among circulating eCB concentrations and psychopathology, particularly in the context of stressful life events, including trauma.

Our group has explored the relationships among circulating eCB concentrations and the *risk* for developing trauma-related psychopathology. Our approach has been to measure circulating eCBs within a few days of traumatic injury and determine whether they are associated with diagnoses of PTSD, depression or chronic pain 6–9 months later. In our first cohort of subjects, *higher* circulating concentrations of AEA (primarily in women) and 2-AG (primarily in individuals from marginalized racial and ethnic groups) at the time of a traumatic injury were associated with *increased* risk for the development of PTSD (deRoon-Cassini et al. 2022). Interestingly, 2-AG and not AEA concentrations were positively associated with depression (Fitzgerald et al. 2021) and chronic pain (Trevino et al. 2022) 6–9 months later.

The aim of the current study was to replicate and expand upon prior work in an independent sample primarily composed of individuals from marginalized racial and ethnic groups and including a greater percentage of females than the original study. Based upon the results of deRoon-Cassini et al. (2022) we hypothesized that higher circulating concentrations of AEA and/or 2-AG at the peri-traumatic period would be positively associated with PTSD symptoms 6–9 months post-injury. We predicted that, similar to the findings in Trevino et al. (2022), that higher circulating concentrations of 2-AG would be positively associated with pain severity 6–9 months postinjury. We also predicted that, similar to Fitzgerald et al. (2021), higher circulating concentrations of 2-AG at the time of injury would predict greater depression 6–9 months post-injury.

## Methods

### Participants

The Institutional Review Board of the Medical College of Wisconsin approved all study procedures. The present study used a sample from a larger longitudinal study (*N* = 199) analyzing the outcomes of traumatic injury (STAR 2.0; Geier et al. 2023). The parent study consisted of adults who were seen in the emergency department (ED) of a Midwestern, urban, Level 1 Trauma Center for a traumatic, acute injury. Patients were excluded from the study if they: (1) were younger than 18 years old; (2) had active suicidal ideation; (3) were on a police hold or discharged to jail; (4) were unable to communicate verbally in either English or Spanish; (5) had a Glasgow Coma Scale of less than 13 (not due to hemorrhagic shock); or (6) had a traumatic brain injury resulting in greater than 24 h of post-traumatic amnesia or more than 30 min of lost consciousness. Because our primary aim was to examine the relationships between peritrauma eCB concentrations and risk for PTSD months later, the present study included all individuals (*n* = 100) from the parent study who had circulating eCB concentrations determined in blood samples taken in the peri-traumatic period and responses for the Clinician-Administered PTSD Scale for DSM-5 (CAPS-5; Weathers et al. 2018) at follow-up. There were no significant differences in age (unpaired t-test: *p* =.42), sex (chi-square test: *p* =.9), race (chi-square test: *p* =.17), or injury severity score (unpaired t-test: *p* =.24) between the individuals in the parent study and those in the present analysis. In some analyses, the present study separated the participants into four groups: White non-Hispanic males (*n* = 13); White non-Hispanic females (*n* = 6); males from marginalized racial and ethnic groups (those endorsing any of the following race or ethnicity categories: Black, Hispanic/Latin-X; American Native or Pacific Islander) (*n* = 47) and females from marginalized racial and ethnic groups (*n* = 34).

### Study procedure

Participants who recently experienced a traumatic injury were recruited either from an in-patient unit (*n* = 74) or the emergency department (*n* = 20), with 6 participants with recruitment location not reported. Those discharged from the emergency department following injury were scheduled to return within 10 days of their injury for their peritrauma visit (M_days_since_injury_ = 4.45, SD = 2.54). Participants recruited from the in-patient unit completed the peri-trauma visit during their hospital stay (M_days_since_injury_ = 3.64, SD = 2.99). During the peri-trauma visit, all participants provided written consent, completed the peri-traumatic assessments, and had a blood draw. All participants returned for a follow-up visit 6–10 months post-injury (M_days_since_injury_ = 205.77, SD = 26.56), at which time they completed the follow-up self-report assessments. A portion of the sample (*n* = 91) had blood drawn again during this follow-up visit. All participants were compensated for their time in the study.

#### Biospecimen collection and analysis

Non-fasting, intravenous blood collection into serum-separating tubes occurred at peri-trauma and follow-up visits. Blood was allowed to clot at room temperature, then was centrifuged at 1400 RPM at 4 °C for 20 min. Serum was pipetted into cryovials and stored at −80 °C until analysis. Circulating eCB concentrations were measured as described previously (Spagnolo et al. 2016; Fitzgerald et al. 2021; deRoon-Cassini et al. 2022), using isotope dilution, liquid chromatography-mass spectrometry to determine the concentrations of the eCBs (AEA and 2-AG). Because the data were log normally but not normally distributed, concentrations of AEA and 2-AG (pMol/mL) were log base 10-transformed before analysis. Recent tetrahydrocannabinol (THC) use was assessed using a urine sample screen at each visit.

#### Clinical and self-report interviews

During the peri-trauma visit, participants self-reported demographic variables (e.g., age, race, education, employment, and relationship status), prior diagnosis of psychiatric disorders, current use of psychiatric medications, and current participation in psychotherapy. Injury Severity Scores (Baker et al. 1974), Glasgow Coma Scores (Teasdale and Jennett 1974; Jain and Iverson 2023), indication of loss of consciousness during the traumatic injury, and mechanism of injury were captured from participants’ medical charts. Self-reported indications of loss of consciousness and injury location were also collected during the peri-trauma visit. During both the peri-trauma and follow-up visit participants were asked about their pain using a rating scale ranging from 1 to 10 as in prior studies (Cleeland and Ryan 1994; Wong and Baker 2013).

##### Depressive symptoms

Depressive symptoms were measured at both the peri-traumatic assessment and follow-up visit using the Depression Anxiety and Stress Scale (DASS-21) depression subscale (Lovibond et al. 1995; Osman et al. 2012). A total severity score for depression was calculated by summing responses to questions relevant to depression and then multiplying that sum by two (Lovibond et al. 1995). Higher scores reflect worse depression symptom severity. The depression subscale within the DASS-21 has high reliability (α = 0.94), and has been found to have a strong correlation with the Beck Depression Inventory (*r* =.79) when given concurrently in a clinical sample (Antony et al. 1998).

Depressive symptoms at the follow up time point were measured utilizing the 10-item version of the Center for Epidemiologic Studies Depression Scale Revised (CESD-R-10) (Eaton et al. 2004; Björgvinsson et al. 2013). The CESD-R-10 has demonstrated a high correlation in community populations with the 20-item version of the questionnaire that has been utilized as a screening tool for depression (Eaton et al. 2004; Björgvinsson et al. 2013; Moon et al. 2017). Each item on this scale is rated by the participant on a 4-point Likert-type scale where 0 represents “not at all/less than 1 day” and 4 represents “nearly every day for 2 weeks”. The CESD-R-10 is scored by summing the responses to all questions, resulting in a total score ranging from 0 to 30 where higher scores indicate higher levels of depression with a suggested cutoff score of 10. The CESD-R-10 has high internal consistency (α = 0.86) and has been found to have good validity (Björgvinsson et al. 2013).

##### Childhood trauma questionnaire

Childhood Trauma was evaluated at the follow-up visit using the Childhood Trauma Questionnaire (Bernstein et al. 1994; Bernstein and Fink 1998). The Childhood Trauma Questionnaire is a 28-item measure where individuals indicate how frequently events occurred in their childhood (e.g., “people in my family looked out for each other”) on a 5-point Likert-type scale ranging from “never true” to “always true.” Some items are reverse coded, then the items are summed into five categories: Emotional Abuse, Physical Abuse, Emotional Neglect, Physical Neglect, and Sexual Abuse. Scores range from 5 to 25 where higher numbers indicate more experiences from childhood that fall within the category. The present study created a sum of these five categories as an index of cumulative childhood maltreatment. For this total score, a suggested cutoff has been 36 in the literature (Vahapoglu et al. 2018).

##### Lifetime trauma exposure

Lifetime trauma exposure was evaluated at the peri-trauma visit using the Life Events Checklist for the Diagnostic and Statistical Manual of Mental Disorders (5th Edition; LEC-5;Weathers et al. 2013; Weis et al. 2022). The LEC-5 is a 17-item measure where individuals indicate their proximity of exposure to various events (e.g., natural disaster, fire, assault). The scale assesses levels of proximity to the trauma, with responses including, “happened to me”, “witnessed it”, “learned about it”, “part of my job”, “not sure”, and “doesn’t apply”. The weighted total scoring procedure was used, in which responses were weighted according to proximity to the trauma exposure (Weathers et al. 2013; Weis et al. 2022). Weighted total scores range from 0 to 102 where higher numbers indicate closer proximity to the event. There are no cutoff scores for the LEC-5 as this measure is primarily used to identify if participants have experienced the listed life events and their proximity.

##### PTSD symptoms

Participants completed the 20-item PTSD Checklist for DSM-5 (PCL-5) at both timepoints to assess self-reported PTSD symptoms (Weathers et al. 2012; Wortmann et al. 2016). This 20-item measure asks participants to rate how bothered they have been in the past month by DSM-5-defined PTSD symptoms (Weathers et al. 2012; Wortmann et al. 2016). At the follow-up assessment, participants were asked to answer the questions based upon the trauma that brought them into the study. The PCL-5 utilizes a 5-point Likert-type scale where 0 indicates “not at all” and 4 indicates “extremely” (Weathers et al. 2012; Wortmann et al. 2016). Ratings are then summed for a total severity score ranging between 0 and 80, where higher scores indicate higher symptom severity (Weathers et al. 2012; Wortmann et al. 2016). Some initial studies have suggested that a cutoff score between 31 and 33 may be indicative of PTSD (Bovin et al. 2016), however, more research is needed in this area. The PCL-5 has high internal consistency (α = 0.75 to 0.95) and good convergent validity (Wortmann et al. 2016).

The CAPS-5 is widely viewed as the gold standard for PTSD diagnostic assessment in research studies (Weathers et al. 2018). The Clinician-Administered PTSD Scale for DSM-5 (CAPS-5; Weathers et al. 2018) was administered at the follow-up visit by trained study staff. This measure is scored by interviewers for intensity and frequency of each DSM-5 symptom utilizing the following anchor points: 0 if the symptom is “absent”, 1 if the symptom is “mild/subthreshold”, 2 if a symptom is “moderate/threshold”, 3 if a symptom being “severe/markedly elevated”, and 4 if a symptom being “extreme/incapacitating” (Weathers et al. 2018). Ratings of intensity and frequency were then merged and summed to calculate a total severity score where higher values indicate more severe symptoms (the range for this sample was 0 to 54), which has high internal consistency (α = 0.88) and good convergent reliability with the PCL-5 (*r* =.66; Weathers et al. 2018). The CAPS-5 has an item where raters indicate whether participants qualify for a diagnosis of PTSD based on DSM-5 criteria, thus, there is no cutoff score per se. For the CAPS-5 administration, participants were instructed to answer questions solely regarding the traumatic injury that brought them into the study.

##### Trauma quality of life

The Trauma Quality of Life Survey (TQOL; Wanner et al. 2015) was administered to measure factors about post-traumatic quality of life across 4 domains (emotional wellbeing, functional engagement, recovery resilience, and peri-trauma). A total score is calculated by summing responses across the domains resulting in a value between 43 and 172, where higher scores indicate higher quality of life. This survey is a self-report survey made up of 43 items with options presented as a Likert scale ranging from “Strongly Disagree” to “Strongly Agree.” The specific questions used to calculate the functional engagement score of this instrument ask whether the individual needs help with driving, walking up stairs, walking on flat surfaces, dressing, bathing/showering, eating, toileting or preparing meals. The lower the score, the more difficulty the individual has with the listed tasks. The TQOL survey has been found to be valid when comparing to scores on the PTSD Checklist – Civilian Version and the Short Form 36 Health Survey Version 2 (Wanner et al. 2015). The revised form of the TQOL score, which was used here, has been validated for use in individuals with a recent traumatic injury and provides an index of the effects of trauma on long-term quality of life (Herrera-Escobar et al. 2022).

## Data analysis

All analyses were conducted in R version 4.3.0; codes are available upon request. Comparisons of continuous variables between peri-trauma and follow-up visits were made using paired samples t-tests. Chi-squared tests were used to compare current psychiatric medication use, current psychotherapy, and THC urine test results between peri-trauma and follow-up visits. Unpaired t-tests or chi-squared tests were used to explore differences in age, sex, racial group, body mass index (peri-trauma and follow-up), mechanism of injury, injury severity score, life events checklist total events (peri-trauma and follow-up), CESD-R-10 total scores, CAPS-5 total severity, THC urine test results (peritrauma and follow-up) in groups separated by whether participants met diagnostic criteria for PTSD at the follow-up timepoint based on the CAPS-5. For all chi-square tests, t-tests, and correlation tests, p-values were corrected, post-hoc, for multiple comparison using the Holm correction (Holm 1979). Pearson correlation analyses were completed between all variables of interest. Following the approach of deRoon-Cassini and colleagues (2022) we used two-way ANOVAs to compare eCB concentrations at both visits between PTSD positive and PTSD negative groups as categorized using CAPS-5 diagnostic criteria at follow-up. Post-hoc one-way ANOVAs were utilized to explore differences between PTSD positive and PTSD negative groups at each time point, followed by the Tukey HSD test which corrected for multiple comparisons. Post-hoc one-way Welch ANOVAs were used to determine differences in eCB concentration at either visit or PTSD symptoms at follow-up were affected by whether participants experienced head trauma or a loss of consciousness from their traumatic injury.

Following the approach of Fitzgerald and colleagues (2021), relationships between peri-trauma eCB concentrations and symptoms at follow-up were assessed using separate hierarchical linear regressions. In step 1, 2-AG and AEA values were entered as predictors of interest. In step 2, the following covariates were added: age, gender, race group (0 = historically marginalized racial/ethnic groups), prior diagnosis of psychiatric disorders (0 = no), current use of psychiatric medications (0 = no), current participation in psychotherapy (0 = no), peri-trauma DASS depression total score, peri-trauma PCL-5 total score, time since injury (days), time since last food intake (hours), and result of urine toxicology screen for THC (0 = negative screen). In step 3 of the model predicting depression, we added post-traumatic distress severity at 6 months (item 27 global severity on the CAPS-5) as a covariate due to the relationship between depression and PTSD (Kessler 1995; Fitzgerald et al. 2021). All continuous variables were grand-mean centered and scaled before included in each hierarchical regression. We imposed a Holm-correction to correct against multiple comparisons of the two outcomes for the prospective and concurrent analyses (CESD-R-10 and CAPS-5; α = 0.0125). Finally, we repeated the hierarchical analyses with early life experiences of trauma included as a covariate using scores from the Childhood Trauma Questionnaire (Bernstein and Fink 1998; see Supplement).

## Results

### Demographics and descriptive statistics

Participant characteristics are reported in Table 1. Participants were largely young to middle-aged adults, 60% were male, and 81% identified as being from marginalized racial and ethnic groups. 50% of the participants completed at least some college. More than half of the participants were employed at the time of injury (61%) and in a committed relationship (55%). The largest number of injuries sustained were from motor vehicle and motorcycle crashes (50%), 28% of the participants experienced an assaultive-type trauma. Based on information collected from participants’ hospital medical charts, 31% of participants experienced a loss of consciousness from their injury, 60% of participants did not, and 9% of participants had this information missing or labeled as unknown in their chart. This is varied from the self-reported data where 54% reported not experiencing a loss of consciousness during the trauma. 38% of participants reported no head trauma occurred; this information was not collected in participants’ medical chart. For all analyses, self-reported data for loss of consciousness and presence of head injury were used.

Loss of consciousness during the traumatic injury had no effect on any eCB serum concentration measurement (peri-trauma AEA (F_3,13.87_ = 0.97, n.s.), peri-trauma 2-AG (F_3, 13.10_ = 0.25, n.s.), follow-up AEA (F_3,8.73_ = 1.74, n.s.), and follow-up 2-AG (F_3, 8.53_ = 0.58)). PTSD symptoms at follow-up were not affected by loss of consciousness during the traumatic injury (F_3, 12.65_ = 0.63, n.s.). Similarly, self-reported experience of head trauma during the injury did not affect any eCB serum concentration measurements (peri-trauma AEA (F_2, 19.64_ = 0.06, n.s.), peri-trauma 2-AG (F_2, 20.91_ = 2.04, n.s.), follow-up AEA (F_2,15.74_ = 0.46, n.s.), and follow-up 2-AG (F_2, 19.14_ = 2.03, n.s.). PTSD symptoms at follow-up also did not vary by experience of head trauma during the traumatic injury (F_2, 18.06_ = 1.61, n.s.).

Mean values for selected clinical data and the primary outcomes at both time points are reported in Table 2. The initial, peri-traumatic assessment and blood draw occurred between 0 and 17 days following the injury (M = 3.7 days, S.D. = 2.9), while the follow-up visit occurred between 180 and 298 days, or 6–10 months after the injury (M = 205 days, S.D. = 26).

At both time points, PCL-5 Total Symptoms scores were highly variable (range 0 to 80). PCL-5 Total Symptoms scores for the aggregate sample increased significantly between peri-trauma and follow-up as did the individual scores for negative mood and cognition, hyperarousal and avoidance (Table 2).

There were significant differences in the PCL-5 Total Symptom Scores among the four defined demographic groups (Figure S1). In accord with what we reported previously in a separate cohort (deRoon-Cassini et al. 2022), White non-Hispanic males had the lowest PCL-5 scores at both time points, and the score did not increase between the peri-traumatic and follow-up assessments. Females from marginalized racial and ethnic groups had the highest mean total PCL-5 score at both time points; above the cut-off of 30 for PTSD diagnosis (Blevins et al. 2015), and the score was significantly greater at follow-up than at the peri-traumatic assessment (paired t(33) = 2.19, *p* =.036).

At the follow-up visit, 26% of the participants met diagnostic criteria for PTSD based upon the CAPS-5 (Table 2). At both time points, DASS depression scores were highly variable, ranging from 0 to 42 and there was no significant change between the time points (Table 2). At follow-up, CESD-R-10 scores ranged from 0 to 27 (M = 10.8, SD = 7.44) and 33 participants (33%) met clinical criteria for Major Depressive Disorder based on symptom severity.

Most participants did not endorse prior psychiatric diagnoses, were not currently using psychiatric medications and were not undergoing psychotherapy; there were no significant changes in these responses between the two visits. 30% of the cohort were urine THC positive at the time of injury; the percentage increased marginally to 38% at the follow-up visit although this was not a statistically significant change.

### Peri trauma serum eCB concentrations are associated with PTSD diagnosis at follow-up

The subset of the participants (*n* = 91) for whom serum concentrations of the eCBs were measured at both time points were divided into two groups using the CAPS-5 assessment at follow-up (i.e., with symptoms consistent with [*n* = 24] and inconsistent with [*n* = 67] a diagnosis of PTSD). A comparison of the participant demographics and outcome measures in each group is provided in Supplemental Table 1. Those with symptoms consistent with a diagnosis of PTSD were more likely to have experienced an assaultive trauma and had greater CESD-R-10 scores at the follow-up assessments. Initial analyses also found that those with symptoms consistent with a diagnosis of PTSD were also significantly younger; were all members of a racial or ethnic marginalized group; and were more likely to have a positive urinary THC result at the peri-traumatic assessment, although these patterns did not survive Holm’s corrections.

**Fig. 1:**
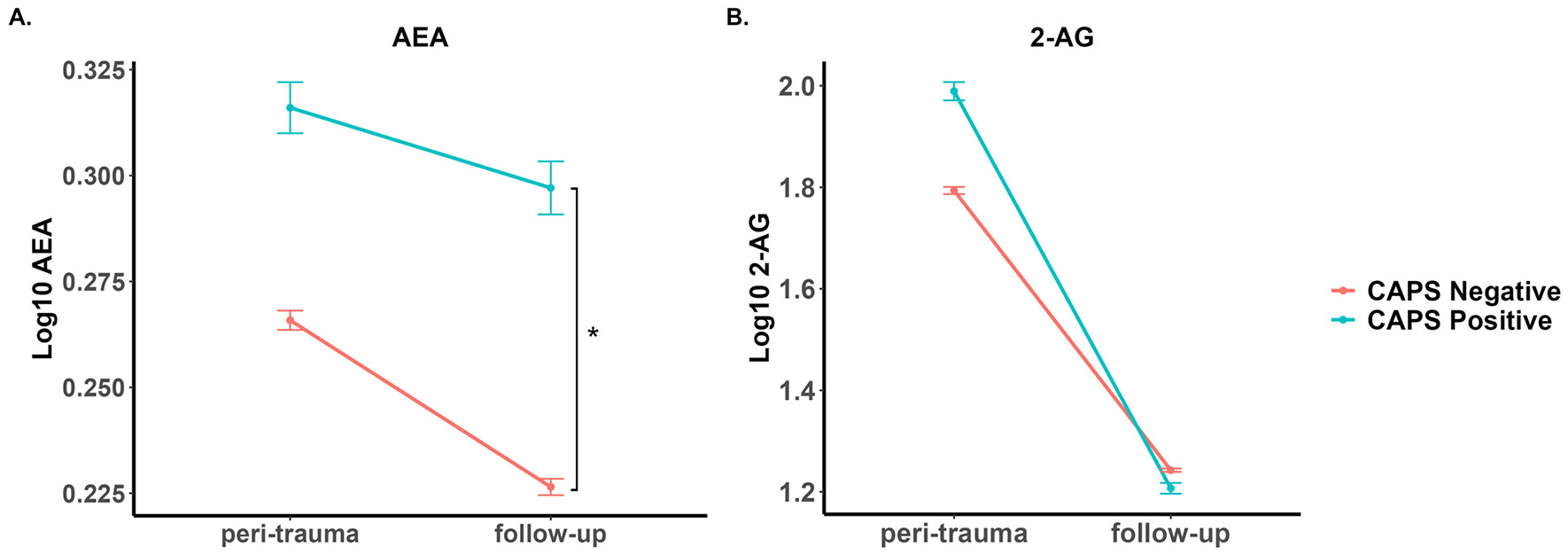
Serum concentrations of AEA and 2-AG were determined within several days of injury (peri-trauma) and 6–10 months later (follow-up). At the follow-up visit, PTSD was diagnosed using the CAPS-5 tool. CAPS Negative (*N* = 67) refers to those without symptoms consistent with a PTSD diagnosis while CAPS Positive (*N* = 24) refers to those with symptoms consistent with a PTSD diagnosis. * *p* <.05 comparing groups indicated by brackets

Concentrations of eCBs in serum obtained at the peri-traumatic and follow-up time points were compared between the CAPS diagnostic groups (Fig. 1). Two-way ANOVA demonstrated no significant effects of PTSD diagnosis criteria (F_1,183_ = 2.73, *n.s.*) or time (F_1,183_ = 0.55, *n.s.*) on serum AEA concentrations. Although the interaction of these main effects was nonsignificant (F_1,183_ = 0.26, *n.s.*), we carried out post-hoc tests on the basis of our findings in the first cohort. Post-hoc comparisons of the values using a one-way ANOVA at each time point revealed serum AEA concentrations at follow-up were higher in individuals with PTSD diagnosis following Tukey HSD test correction (F_1,89_ = 3.99, *p* <.05, p_adj_ < 0.05; Fig. 1A).

Two-way ANOVA of the serum 2-AG data demonstrated significant effects of both PTSD (F_1,183_ = 4.09, *p* <.05) and time (F_1,183_ = 31.7, *p* <.0001) as well as a significant interaction (F_1,183_ = 4.10, *p* <.05). However, post-hoc one-way ANOVAs revealed 2-AG concentrations did not differ by PTSD diagnosis at either time point (peri-traumatic: F_1,98_ = 2.85, *n.s.*; follow-up: F_1,89_ = 0.35, *n.s.*; Fig. 1B).

**Fig. 2:**
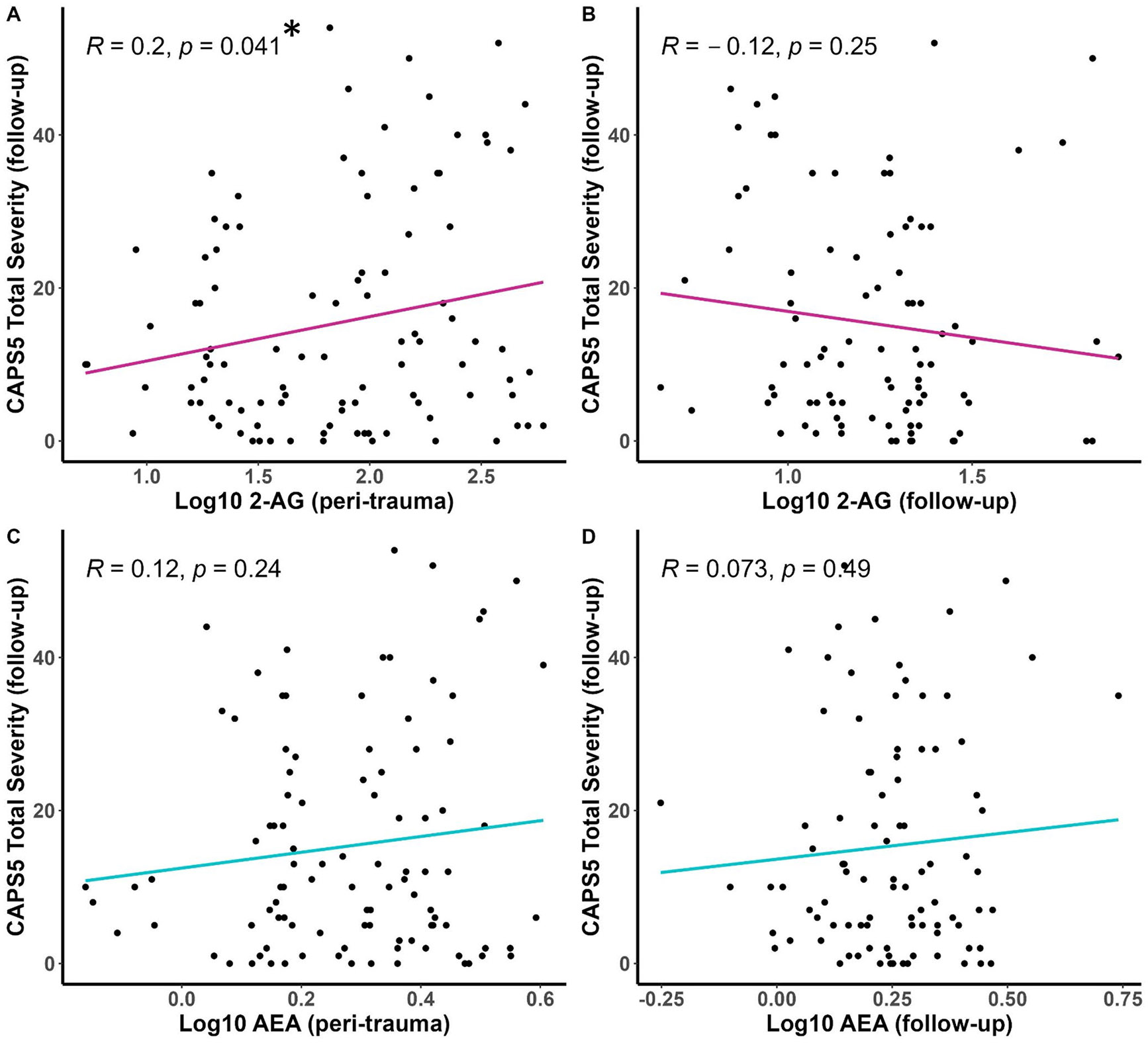
CAPS-5 total severity scores were measured at follow-up (6–10 months after the injury) and serum eCB concentrations were measured in blood harvested within several days of the injury (peri-trauma, panels **A** and **C**) or in blood harvested at the follow-up visit (follow-up, panels **B** and **D**). Pearson’s R values and associated *p* values are reported on each panel. * *p* <.05 for the relationship in panel **A**

However, correlational analyses using each eCB and the CAPS-5 scores as continuous variables support a positive and significant relationship between the serum concentration of 2-AG at the peri-traumatic assessment and CAPS-5 PTSD total severity score (Fig. 2A). There were no significant correlations between the CAPS-5 severity and AEA concentrations at either time point (Fig. 2C, D) or 2-AG concentrations at follow-up (Fig. 2B). The same correlational analyses were carried out separately in male and female participants (Supplemental Fig. 2). The positive relationship between peri-traumatic serum 2-AG concentrations and the total CAPS-5 score at follow-up seen in the entire group of participants was replicated in male (Supplementary Fig. 2A) but not female participants (Supplementary Fig. 2E). The male participants exhibited a positive relationship between serum AEA concentrations measured at follow-up and the total severity CAPS-5 score (Supplementary Fig. 2D) while female participants exhibited a negative relationship between peri-traumatic serum 2-AG concentrations and the total severity CAPS-5 score. However, none of these correlations survived correction for multiple comparisons.

Results of hierarchical regression analyses (Table 3) predicting CAPS-5 severity scores at follow-up was not significant (*F*_2, 87_ = 2.67, *n.s.*; R^2^ = 0.058) in Step 1 where only the eCBs were used as variables. With the addition of covariates in Step 2, the overall model was significant (*F*_14, 75_ = 6.059, *p* <.025; R^2^ = 0.531), and higher serum 2-AG concentration was significantly related to greater PTSD symptoms (B = 0.239, *p* <.025; Table 3), however this relationship did not survive correction for multiple comparisons. Of the added covariates in step 2, only peri-trauma PCL-5 symptom severity was a significant predictor of CAPS-5 severity scores at follow-up (B = 0.602, *p* <.001).

### Peri trauma serum 2-AG concentrations are associated with pain severity at follow-up

**Fig. 3:**
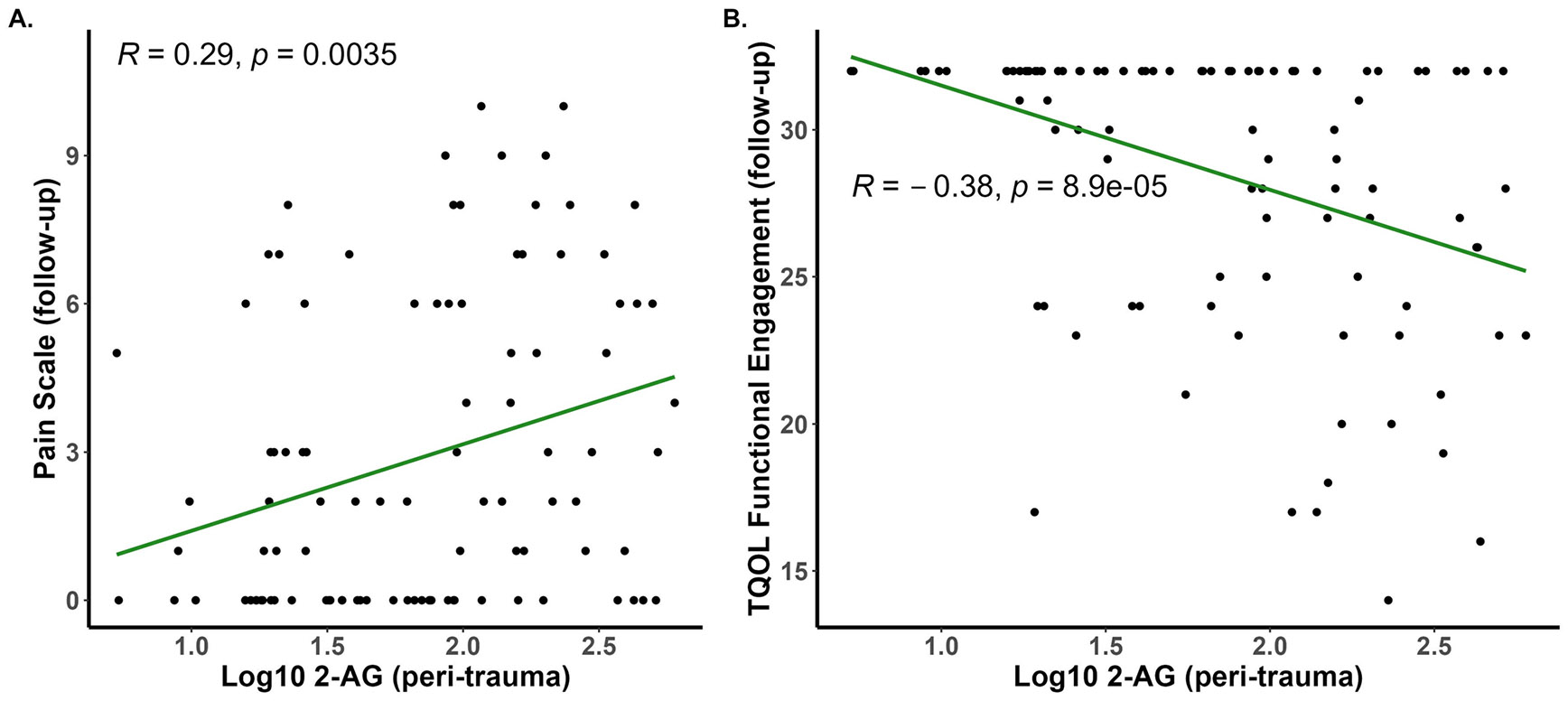
Pain severity on a 10-point scale (**A**) or functional engagement subscores from the TQOL (**B**) were measured at follow-up (6–10 months after the injury) and serum eCB concentrations were measured in blood harvested within several days of the injury (peri-trauma). Pearson’s R values and associated *p* values are reported on each panel

Correlational analyses demonstrate a significant, positive correlation between pain severity at follow-up and the peri-traumatic serum concentration of 2-AG (Fig. 3A). Positive correlations were also seen when data from male and female participants were analyzed separately (Supplementary Fig. 3A). Hierarchical analysis (Table 3) revealed that peri-trauma 2-AG concentrations positively predicted pain severity at follow-up (B = 1.040, *p* <.025) in Step 1 and the model as a whole was significant (F_2, 86_ = 6.593, *p* <.025, R^2^ = 0.133). With the addition of covariates in Step 2, the model remained significant (F_14, 74_ = 3.114, *p* <.025, R^2^ = 0.371) and peri-trauma serum 2-AG concentrations continued to significantly, positively predict pain at follow-up (B = 0.860, *p* <.025). Of the added covariates in step 2, only peri-trauma PCL-5 symptom severity was a significant predictor of pain severity at follow-up (B = 1.126, *p* <.025).

### Peri trauma serum 2-AG concentrations are associated with functional engagement component of TQOL at follow-up

A significant, positive correlation between the functional engagement component of the TQOL instrument measured at the follow-up assessment and the peri-traumatic serum concentration of 2-AG was found (Fig. 3B). This relationship was seen in both male and female participants (Supplementary Fig. 3B). In accord with the correlational results, hierarchical analysis demonstrated that peri-trauma 2-AG concentration was highly, negatively related to the functional engagement component of the TQOL (B = −0.365, *p* <.001) and the model as a whole was significant (F_2, 87_ = 8.242, *p* <.025, R^2^ = 0.159) in Step 1. With the addition of covariates in Step 2, 2-AG concentration continued to negatively predict functional engagement in the TQOL instrument (B = −0.384, *p* <.01) and females were at greater risk for low functional engagement than males (B = −0.503, *p* <.05). The model as a whole in Step 2 was significant (F_14, 75_ = 2.660, *p* <.025, R^2^ = 0.332). There were no other significant correlational results between other components of TQOL and either eCB measured at the time of trauma; however, 2-AG concentrations and the emotional well-being component of the TQOL both assessed at follow-up were positively correlated (Supplemental Fig. 4).

### Serum eCB concentrations are not associated with measures of depression at follow-up

Results of the hierarchical regression of peri-trauma eCB concentration and CESD-R-10 total scores at follow-up showed AEA and 2-AG concentrations were not related to depressive symptoms in Step 1, and the overall model was not significant (Supplementary Table 2). With the addition of covariates in Step 2, the overall model was significant, but neither AEA nor 2-AG concentrations were significantly related to depressive symptoms; peri-trauma depression measured by the DASS-21 (B = 0.366, *p* <.01) was the only significant predictor and this relationship survived correction for multiple comparisons. Step 3 accounted for concurrent symptoms of PTSD, captured in the CAPS distress score. In this model, both peri-trauma depression (B = 0.345, *p* <.01) and concurrent distress (B = 0.423, *p* <.01) were significant predictors of depression. Neither 2-AG nor AEA were significant predictors in Step 3 following correction for multiple comparisons. However, AEA positively predicted depressive symptoms before this correction (B = 0.177, *p* <.05).

### Correlational studies of circulating eCB concentrations and other measures

Correlational analyses were used to explore the relationships between the circulating eCBs and other continuous variables (Supplementary Fig. 4). Age was positively correlated with concentrations of 2-AG at follow-up (*r* =.33, *p* <.001). There was a significant, positive correlation between serum AEA and 2-AG concentrations in the follow-up sample (*r* =.34, *p* <.001). Participant injury severity scores at the peri-trauma assessment were positively correlated with peri-trauma 2-AG concentration (*r* =.29, *p* <.05) but negatively correlated with 2-AG concentration at the follow-up assessment (*r* = −.27, *p* <.05). Peri-trauma BMI was positively correlated with follow-up 2-AG concentrations (*r* =.24, *p* <.05). At the peri-trauma assessment, serum concentrations of AEA were positively correlated with systolic blood pressure (*r* =.39, *p* <.001) and with time since last meal (*r* =.39, *p* <.001). Time since last meal at the follow-up visit was negatively correlated with 2-AG concentration (*r* = −.23, *p* <.05), but positively correlated with AEA concentration (*r* =.25, *p* <.05). None of these relationships between circulating eCB concentrations and other measures remained significant following the Holm correction. Components of the PCL-5, CAPS, TQOL, CTQ, and pain scoring instruments were highly correlated among one another.

## Discussion

The goal of this study was to determine whether the relationships between circulating eCB concentrations and risk for the development of long-term consequences from a previous cohort of survivors of traumatic injury (Fitzgerald et al. 2021; deRoon-Cassini et al. 2022; Trevino et al. 2022) replicated in a second cohort. The results of the first cohort demonstrated circulating AEA and 2-AG concentrations in the peri-traumatic period were significantly, positively associated with the occurrence and severity of PTSD in women and individuals from marginalized ethnic and racial groups, respectively, six-ten months after the index injury (deRoon-Cassini et al. 2022). In addition, circulating 2-AG concentrations were positively associated with a diagnosis of depression (Fitzgerald et al. 2021) and severity of chronic pain (Trevino et al. 2022) 6–10 months later. Like those studies, this study was carried out in traumatically injured individuals who presented for treatment at an urban, level one trauma center. Serum eCB concentrations were measured in the peri-traumatic period and at a followup assessment (6–10 months later) when PTSD, chronic pain, depression and TQOL were assessed. The conclusions of the initial study that circulating 2-AG concentrations in the peri-traumatic period were positively associated with PTSD and chronic pain were replicated in the current cohort. While the associations did not survive correction for multiple comparisons, similar to the initial study, there was some evidence of 2-AG concentrations’ positive association with PTSD in the current cohort. In addition, we found the TQOL Functional Engagement measure determined at follow-up was highly, negatively, associated with serum 2-AG concentrations in the peri-traumatic period. On the other hand, associations between 2-AG concentrations and depression and AEA concentrations and PTSD were not replicated in this cohort.

There are notable similarities and differences between the demographics of the present study sample and the previous study cohort. The present cohort included a significantly greater percentage of individuals identifying as being from racially/ethnically marginalized groups (81% compared to 54% in the previous cohort) and a higher proportion of females (43% compared to 31% in the previous cohort). These differences are notable as racial/ethnic status was identified as an important modulator of the association between circulating eCBs and risk for PTSD (deRoon-Cassini et al. 2022) and contributed significantly to the risk for depression (Fitzgerald et al. 2021). In addition, sex differences in the relationship between circulating eCBs and PTSD (deRoon-Cassini et al. 2022) and chronic pain (Trevino et al. 2022) were seen in the first cohort, with females being at increased risk in both studies.

Consistent with findings in the previous cohort (deRoon-Cassini et al. 2022), female members of racially/ethnically marginalized groups had the highest PTSD symptom severity both at the time of the index trauma and 6–10 months later. Peri-traumatic serum concentrations of both AEA and 2-AG were higher in individuals who ultimately were diagnosed with PTSD at follow-up in both cohorts as well. Interestingly, however, the relationship was stronger for AEA in the previous cohort while 2-AG, and not AEA, was significantly, positively associated with PTSD severity in the current study. In both cohorts, peri-traumatic serum 2-AG concentrations and PTSD severity at follow-up *were* significantly associated in the subset of participants from ethnically/racially marginalized groups.

Prior work indicates that there may be an increased risk for the development of depression (White et al. 2007; Ali et al. 2017) and PTSD (Alegría et al. 2013; Berger and Sarnyai 2015; Sibrava et al. 2019; Bird et al. 2021) following traumatic injury in racially/ethnically marginalized individuals. Increased experiences of racism and discrimination involve long-term exposure to stress impacting biological systems (e.g., the HPA Axis; Berger and Sarnyai 2015) which adds to traumatic stress (Sibrava et al. 2019; Brooks Holliday et al. 2020; Bird et al. 2021). Similarly, other external factors (e.g., neighborhood safety, income) that add to levels of stress alone are associated with an increased number of traumatic events (Ali et al. 2017; Tomas et al. 2025). A recent analysis found that, even when controlling for other environmental factors, experiences of racism were associated with screening positive for PTSD (Brooks Holliday et al. 2020). A separate study found that when controlling for environmental factors Black/African American individuals were more likely to endorse higher rates of depression following Hurricane Katrina than White individuals; however this finding was confounded by social support, preexisting vulnerabilities, and hurricane-related stressful exposures (Ali et al. 2017). The current results highlight the need for work focusing on the impact of contextual factors (e.g., experiences of discrimination, chronic stress) on circulating eCB concentrations and how this contributes to subsequent development of psychopathology following a traumatic injury (Fani et al. 2021; Bird et al. 2021; Roeckner et al. 2022).

In accordance with results in the previous cohort (Trevino et al. 2022), we identified a significant positive association between peri-traumatic serum 2-AG concentrations and severity of pain 6–10 months later. While in the previous study, female sex was also an important determinant of pain severity, hierarchical regression analyses in the current study did not identify sex as a contributor to chronic pain risk. Rather, correlational analyses demonstrated a significant, positive association between peri-trauma 2-AG concentrations and chronic pain in both males and females. Our hierarchical analysis also found that peri-trauma PCL-5 scores were positively associated with chronic pain, results that are consistent with evidence that chronic pain and PTSD are often co-morbid (Siqveland et al. 2017). A novel finding in the present study is that peri-trauma 2-AG concentrations were negatively associated with the functional engagement component of the TQOL score. It is possible that the relationship between circulating 2-AG and functional TQOL is secondary to the relationship between circulating 2-AG and pain, given that chronic pain was highly, negatively associated with the functional TQOL (Supplemental Fig. 4).

We explored the relationship between peri-trauma 2-AG serum concentration and depressive symptoms at followup. Our previous study found that higher concentrations of 2-AG predicted greater depression 6–9 months post-injury (Fitzgerald et al. 2021), this was not seen in the current cohort. These discrepancies underscore the complexity of eCB involvement in depression, as reflected in the inconsistent literature in this area (Hill et al. 2009; Ibarra-Lecue et al. 2018; Stensson et al. 2020; Fitzgerald et al. 2021). Depression rates during the present study’s peri-trauma and 6–10 month time points are notably higher than those reported in Fitzgerald et al. (2021); it is possible that circulating eCBs are associated with less severe depression symptoms. Alternatively, circulating eCB concentrations may indicate risk for depression differently at varying times after injury. Further work could follow how the eCB system changes over time with the development and recurrence of depressive symptoms.

These data, together with our prior study, support the hypothesis that circulating eCBs, particularly 2-AG, are biomarkers of the risk for developing long-term consequences following a traumatic injury. While peri-trauma circulating 2-AG concentrations are highly elevated in all injured individuals, it is our current hypothesis that factors related to the injury (i.e. its physical or psychological severity; degree of pain), prior traumatic experiences, and demographics (i.e. age, sex) may all contribute to the degree to which 2-AG is elevated. As these are factors that also individually influence the long-term physical and psychological sequelae of traumatic injury, it is possible that circulating 2-AG concentrations are an integration of these factors and so serve as biomarkers of risk but 2-AG per se does not contribute causally to the outcomes.

On the other hand, it could be excessive elevation of 2-AG in the circulation reflects excessive 2-AG/CB1R signaling in the brain, which is known to occur with chronic stress and result in CB1R downregulation (Hillard 2014). Preclinical studies demonstrate that loss of CB1R signaling in the CNS is associated with enhanced anxiety, poor regulation of fear extinction, excessive pain and depressed mood (deRoon-Cassini et al. 2020). Thus, the elevation of 2-AG that occurs in response to traumatic injury could put individuals at risk for long-term, negative outcomes as a result of CB1R downregulation and, therefore, loss of the eCB/CB1R stress buffer. Given that discrimination, racism and oppression are chronic stressors (Sibrava et al. 2019; Brooks Holliday et al. 2020; Bird et al. 2021), it is possible that this circumstance is particularly relevant for individuals who have experienced longstanding chronic stress due to sociological conditions. An important consideration for this hypothesis is the time course of the elevation in 2-AG following traumatic injury. While we did not examine the time course of 2-AG elevation in this study, studies of acute stress in healthy subjects suggest that the effects of acute stress on circulating eCB concentrations are short lived (Choukèr et al. 2010). Further exploration of how the CNS eCB system changes acutely and dynamically over time following an injury, particularly whether CB1R density is altered, will shed light on these possibilities and is needed to inform intervention.

A unique aspect of the eCB signaling system is that CB1R activity is regulated by two endogenous ligands. While both AEA and 2-AG are thought to regulate presynaptic transmitter release, it is thought that 2-AG is a fast-on, fast-off ‘phasic’ signal while AEA behaves as a ‘tonic’ signal (Morena et al. 2016). Differential responsiveness of AEA and 2-AG to interventions such as psychological stress (Dlugos et al. 2012), exercise (Koltyn et al. 2014), and exposure to a negative stimulus (Weisser et al. 2022) in healthy humans suggest that circulating concentrations of both eCBs can be elevated in response to stressors (Gowatch et al. 2024) and exercise (Desai et al. 2022). Although more studies need to be done, current data suggest that AEA concentrations are more sensitive to psychological stress (Gowatch et al. 2024), while 2-AG concentrations are more affected by time of day (Hanlon et al. 2015) and physical trauma (data herein). A recent study investigating how changes in eCB concentrations relate to behavioral responses to threats found that increased AEA concentrations are associated with stronger acquisition of fear memories and activation of the right amygdala while high concentrations of 2-AG were associated with fear expression and impairment of fear extinction and activation of the left hippocampus (Weisser et al. 2022). Thus, available data are beginning to elucidate the specific roles of 2-AG and AEA in biological response to stress and trauma.

There are several limitations of the present study. First, the study assessed circulating eCB concentrations and psychological measures at only two points in time. The trajectory of change in 2-AG concentrations between the trauma and recovery time points is not known and is likely an important factor. Second, the relationship between circulating eCB concentrations and brain eCB/CB1R signaling is not known. Simultaneous measurement of circulating eCB concentrations and neuroimaging is beginning to shed light on this question. One recent study examined neuroimaging associations with 2-AG concentrations during a fear generalization task in mice and human subjects concluded that circulating eCBs parallel activity within the brain in response to stress (Rosas-Vidal et al. 2024), suggesting overlap in the mechanisms that regulate eCB concentrations in the periphery and brain. Third, we were not able to explore the relationship of genetic polymorphisms with circulating eCB concentrations and symptom severity although findings have suggested that certain polymorphisms can increase the risk for the development of symptoms after a trauma (Fitzgerald et al. 2021; deRoon-Cassini et al. 2022). Fourth, there was a wide range of mechanisms of injury in this study sample. Future studies with greater numbers of participants could examine whether injury type is an important determinant of 2-AG concentrations and risk. Fifth and finally, we do not have data from the CAPS-5 or CESD-R-10 at the peri-trauma time point. While we had other assessments for depressive (DASS-21) and PTSD (PCL-5) symptoms at the peri-trauma visit, consistency in measures across time points could have offered opportunity to explore whether eCB concentration predicts symptom onset or persistence. Future studies should strive to explore this relationship with consistent measures of PTSD and depression, as well as use more frequent assessment points to better track eCB concentration changes following trauma.

Overall, the present study expands upon prior investigations (Fitzgerald et al. 2021; deRoon-Cassini et al. 2022; Trevino et al. 2022), exploring the relationship between circulating eCB concentrations and adverse symptoms after a traumatic injury. These studies demonstrate that it is important to include a diverse population in studies of PTSD, chronic pain and other negative outcomes from injury and support the hypothesis that the eCBs, particularly 2-AG, in the circulation is related to long-term risk. These findings could potentially inform targeted intervention efforts in individuals from marginalized racial and ethnic groups as they are more at-risk for the development of PTSD and chronic pain.

## Supplementary Material

Supplement

**Supplementary Information** The online version contains supplementary material available at https://doi.org/10.1007/s00213-025-06837-4.

## Figures and Tables

**Table 1:** Characteristics of study participants

	Overall (*N* = 100)
Sex	
Male	60 (60.0%)
Age (years)	
Mean (SD)	38.2 (14.6)
Median [Min, Max]	36.3 [18.2, 76.5]
Ethnicity/Race	
American Indian/Alaska Native	1 (1.0%)
Asian	2 (2.0%)
Black or African American	53 (53.0%)
Hispanic or Latino	24 (24.0%)
Native Hawaiian or Other Pacific Islander	1 (1.0%)
White	19 (19.0%)
Education completed	
Less than high school	15 (15.0%)
Graduated high school	35 (35.0%)
Some college	33 (33.0%)
College graduate	13 (13.0%)
Advanced degree (master’s degree or higher)	4 (4.0%)
Currently employed?	
Yes	61 (61.0%)
Missing	1 (1.0%)
In a committed relationship?	
Yes	55 (55.0%)
Mechanism of Injury	
Motor vehicle crash	42 (42.0%)
Gun shot	21 (21.0%)
Stab	3 (3.0%)
Fall	11 (11.0%)
Pedestrian struck	6 (6.0%)
Motorcycle crash	8 (8.0%)
Crush injury	1 (1.0%)
Other, assaultive	4 (4.0%)
Other, non-assaultive	4 (4.0%)
Loss of Consciousness During Trauma - Medical Chart	
No	60 (60%)
Yes	31 (31%)
Missing/Unknown	9 (9%)
Loss of Consciousness During Trauma - Self Report	
No	54 (54%)
Yes (Witnessed)	13 (13%)
Suspected (Not Witnessed)	29 (29%)
Unknown	4 (4%)
Head Trauma or Sudden Acceleration/Deceleration	
None	38 (38%)
Struck by/against (contact with head/face)	53 (53%)
Whiplash (forceful jolt to head without contact)	8 (8%)
Missing	1 (1%)
Assaultive Trauma	
Yes	28 (28%)
No	72 (72%)
Injury Severity Score (ISS)	
Mean (SD)	13.1 (9.53)
Median [Min, Max]	10 [1.00, 43.0]
Missing	21 (21.0%)
Life Events Checklist Weighted Total Score (Peri-Trauma)	
Mean (SD)	20.7 (12.6)
Median [Min, Max]	19.0 [0, 68.0]

**Table 2:** Descriptive statistics for study variables of interest

	Peri-Trauma	Follow-Up	T-Test or Chi- Squared Test	Holm Adjusted *p*-value
Days Since Injury			-	
Mean (SD)	3.70 (2.86)	205 (26.1)		
Median [Min, Max]	3.00 [0, 17.0]	193 [180, 298]		
Systolic Blood Pressure			t = 2.81 **	*p* <.05 *
Mean (SD)	126 (18.4)	132 (16.7)		
Median [Min, Max]	124 [85.0, 174]	128 [104, 180]		
Missing	0 (0%)	6 (6.0%)		
Body Mass Index			t = −2.84 **	*p* <.05 *
Mean (SD)	32.3 (9.12)	30.4 (10.8)		
Median [Min, Max]	31.4 [16.8, 68.5]	29.6 [0, 69.4]		
Missing	2 (2.0%)	8 (8.0%)		
Time Between Blood Draw and Last			t = −1.29	n.s.
Food Intake (h)				
Mean (SD)	10.1 (17.9)	7.40 (6.95)		
Median [Min, Max]	2.91 [0, 99.5]	3.63 [0.12, 26.8]		
Missing	4 (4.0%)	7 (7.0%)		
PCL-5 Total Symptoms			t = 3.00 **	*p* <.05 *
Mean (SD)	23.6 (20.9)	28.6 (23.2)		
Median [Min, Max]	17.0 [0, 74.0]	25.5 [0, 80.0]		
PCL-5 Reexperiencing			t = 0.07	n.s.
Mean (SD)	6.66 (6.82)	6.70 (6.60)		
Median [Min, Max]	4.50 [0, 20.0]	4.50 [0, 20.0]		
PCL-5 Avoidance			t = 3.19 **	*p* <.05 *
Mean (SD)	2.35 (2.93)	3.30 (3.05)		
Median [Min, Max]	1.00 [0, 8.00]	3.00 [0, 8.00]		
PCL-5 Negative Mood			t = 2.49 *	n.s.
Mean (SD)	7.99 (7.45)	9.82 (8.70)		
Median [Min, Max]	6.00 [0, 27.0]	8.50 [0, 28.0]		
PCL-5 Hyperarousal			t = 3.88 ***	*p* <.01 **
Mean (SD)	6.62 (6.13)	8.77 (6.82)		
Median [Min, Max]	5.00 [0, 22.0]	9.00 [0, 24.0]		
CAPS Total Severity (Mean Impute)			-	
Mean (SD)	-	15.4 (14.5)		
Median [Min, Max]	-	10 [0, 54.0]		
CAPS-based PTSD Diagnosis			-	
Yes	-	26 (26.0%)		
DASS 21 Depression			t = −1.05	n.s.
Mean (SD)	10.7 (11.8)	9.53 (11.5)		
Median [Min, Max]	6.00 [0, 42.0]	4.00 [0, 42.0]		
Missing	1 (1.0%)	1 (1.0%)		
CESD Total Score	-			
Mean (SD)	-	10.8 [7.44]		
Median [Min, Max]	-	10 [0, 27.0]		
Current Psych Medication Use			χ^2^ = 0.00	n.s.
No	79 (79.0%)	79 (79.0%)		
Yes	21 (21.0%)	20 (20.0%)		
Missing	0 (0%)	1 (1.0%)		
Current Psychotherapy			χ^2^ = 0.05	n.s.
Yes	11 (11.0%)	13 (13.0%)		
Urine THC			χ^2^ = 5.58	n.s.
Negative	64 (64.0%)	55 (55.0%)		
Positive	30 (30.0%)	38 (38.0%)		
Missing	6 (6.0%)	7 (7.0%)		

**Table 3:** Hierarchical regression results: Peri-trauma serum endocannabinoid concentrations and CAPS-5 severity, pain scores and functional engagement TQOL at follow-up

	CAPS-5 Severity		Pain Score		Functional Engagement TQOL	
		
Predictor	Step 1	Step 2	Step 1	Step 2	Step 1	Step 2
Serum AEA – Peri-trauma	0.137	0.182	0.522	0.279	−0.164	−0.201
Serum 2-AG – Peri-trauma	0.198	**0.239** **	**1.040** **	**0.860** **	**−0.365** ***	**−0.384** ***
Age – Peri-trauma		−0.003		0.071		−0.079
Sex (0 = Male)		0.221		0.449		**−0.503** *
Race (0 = historically racially/ethnically marginalized)		−0.202		−1.784		0.432
Current Psychiatric Medication (0 = No)		−0.13		1.671		−0.388
Current Psychotherapy (0 = No)		−0.023		−1.616		0.641
Previous Psychiatric History (0 = No)		0.388		−1.156		−0.097
DASS Depression - Peri-trauma		−0.092		−0.120		0.181
PCL-5 Total - Peri-trauma		**0.602** ***		**1.126** **		−0.268
Days since injury – Peri-trauma		0.131		0.190		−0.079
Hours since food intake – Peri-trauma		−0.156		−0.130		0.026
Urine THC - Peri-trauma (0 = negative)		0.031		0.360		−0.015
LEC Weighted total – Peri-trauma		0.003		0.176		0.014
Constant	0	−0.174	**2.949** ***	**3.186** ***	0	0.197
Observations	90	90	89	89	90	90
R2	0.058	0.531	0.133	0.371	0.159	0.332
Adjusted R2	0.036	0.443	0.113	0.252	0.140	0.207
Residual Std. Error	0.982 (df = 87)	0.746 (df = 75)	3.013 (df = 86)	2.768 (df = 74)	0.927 (df = 87)	0.890 (df = 75)
F Statistic	2.671 (df = 2; 87)	6.059** (df = 14;75)	**6.593** ** (df = 2; 86)	**3.114** ** **(df = 14; 74)**	**8.242** ** **(df = 2; 87)**	**2.660** ** **(df = 14; 75)**

*TQOL* Total Quality of Life, *AEA N*-Arachidonoylethanolamine, *2-AG* 2-Arachidonoylglycerol, *DASS* Depression Anxiety and Stress Scale, *PCL-5* PTSD Checklist for DSM-5, *THC* Tetrahydrocannabinol, *LEC* Life Events Checklist for the Diagnostic and Statistical Manual of Mental Disorders, *CAPS-5* Clinician-Administered PTSD Scale for DSM-5; All values shown are unstandardized coefficients unless otherwise stated. * *p* <.05; **p <.025, ***p <.01. Note that the significant relationship between 2-AG and CAPS-5 Severity did not survive Holm’s correction for multiple comparisons. Holm’s correction for multiple comparisons was not explored in the pain or functional engagement models

## Data Availability

The data used in the present study is publicly available through NIH; the code for all analyses is available upon request to the corresponding author.
