# Supplementary material for: Relationships among peri-traumatic circulating endocannabinoids and long-term, negative outcomes following traumatic injury
Source: Psychopharmacology (Berl). Author manuscript; Available in PMC 2026 Feb 26. (PMC12904968; doi:10.1007/s00213-025-06837-4)
Supplement: Supplement [file NIHMS2142601-supplement-Supplement.docx]

**
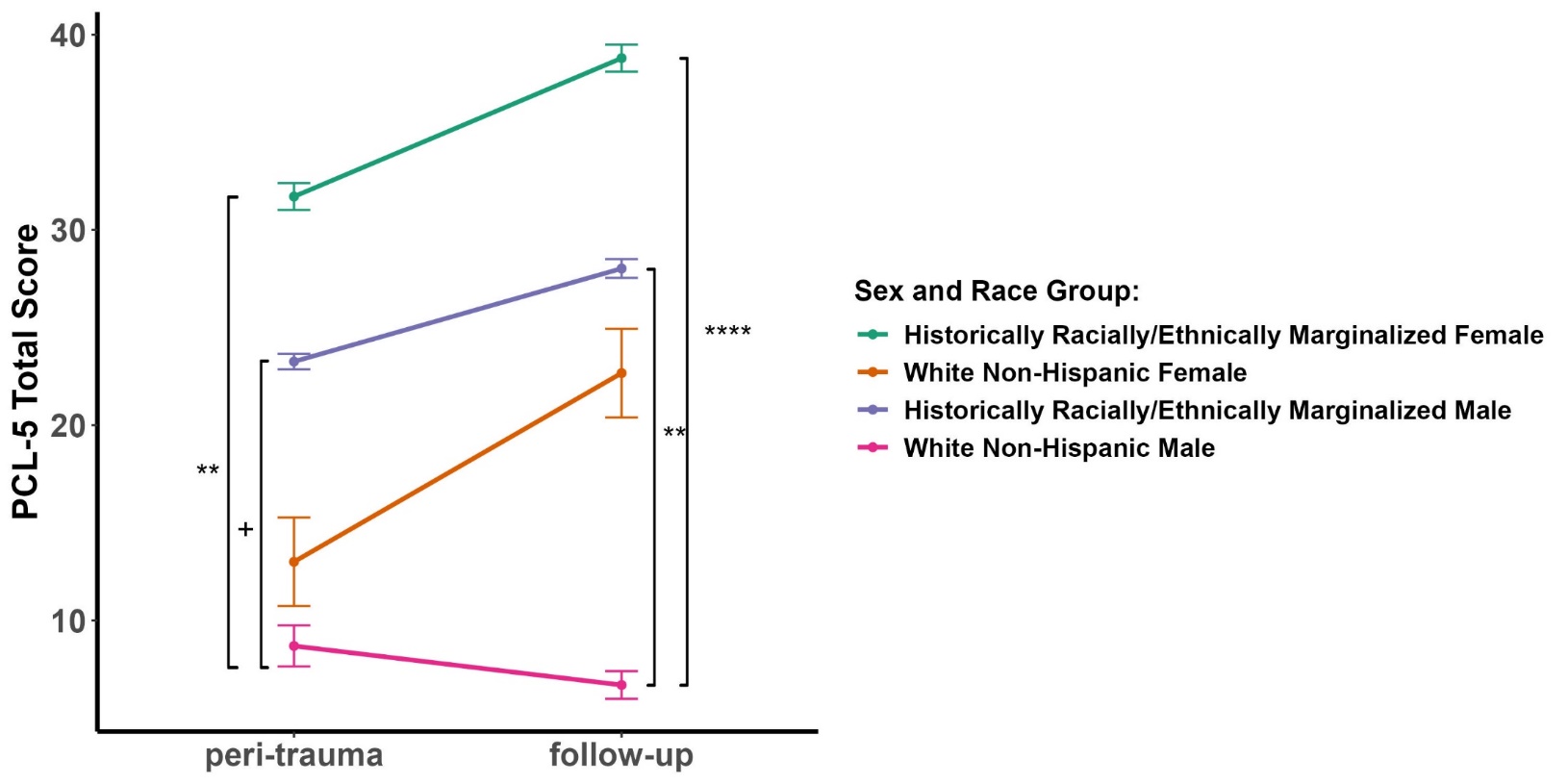
**

**Supplemental Figure 1.** PCL-5 total scores were measured in all participants at the peri-trauma period (within several days of the injury) and at follow-up (6-10 months after the injury). Participants were instructed to reflect only on the index injury to answer the questions at follow-up. The numbers in each group: historically racially/ethnically marginalized females (34); racial/ethnic marginalized males (47); white, non-Hispanic females (6); and white, non-Hispanic males (13). + p<0.1; ** p<0.01; **** p<0.001 comparing groups indicated by brackets.

**
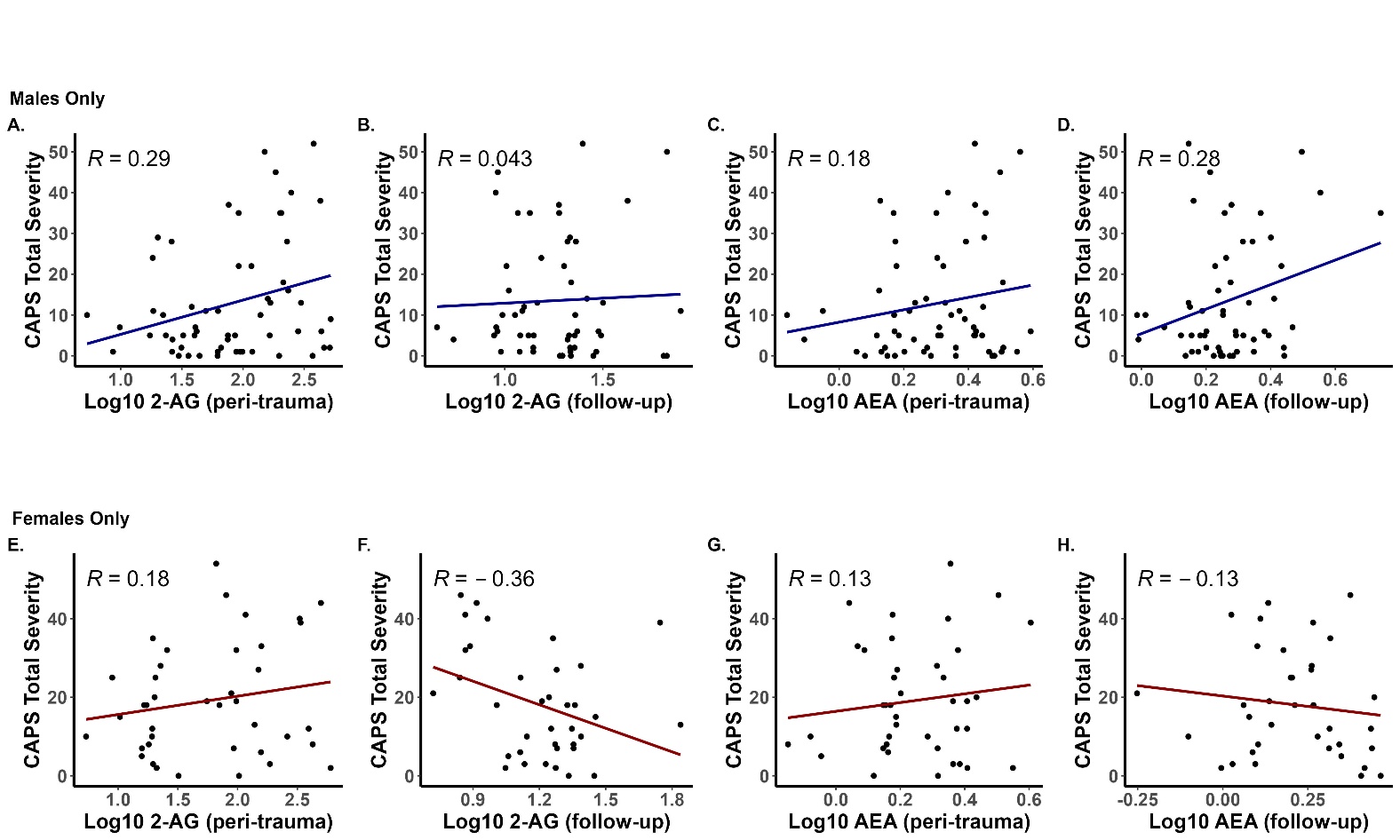
**

**Supplemental Figure 2.** CAPS-5 total severity scores were measured at follow-up (6-10 months after the injury) and serum eCB concentrations were measured in blood harvested within several days of the injury (peri-trauma) and in blood harvested at the follow-up visit (follow-up). Panels A-D includes data from participants identifying as female (n = 40) and Panels E-H includes data from participants identifying as male (n = 60). Pearson’s R values and associated are reported on each panel, p-values are not reported as no correlation survived correction for multiple comparisons.


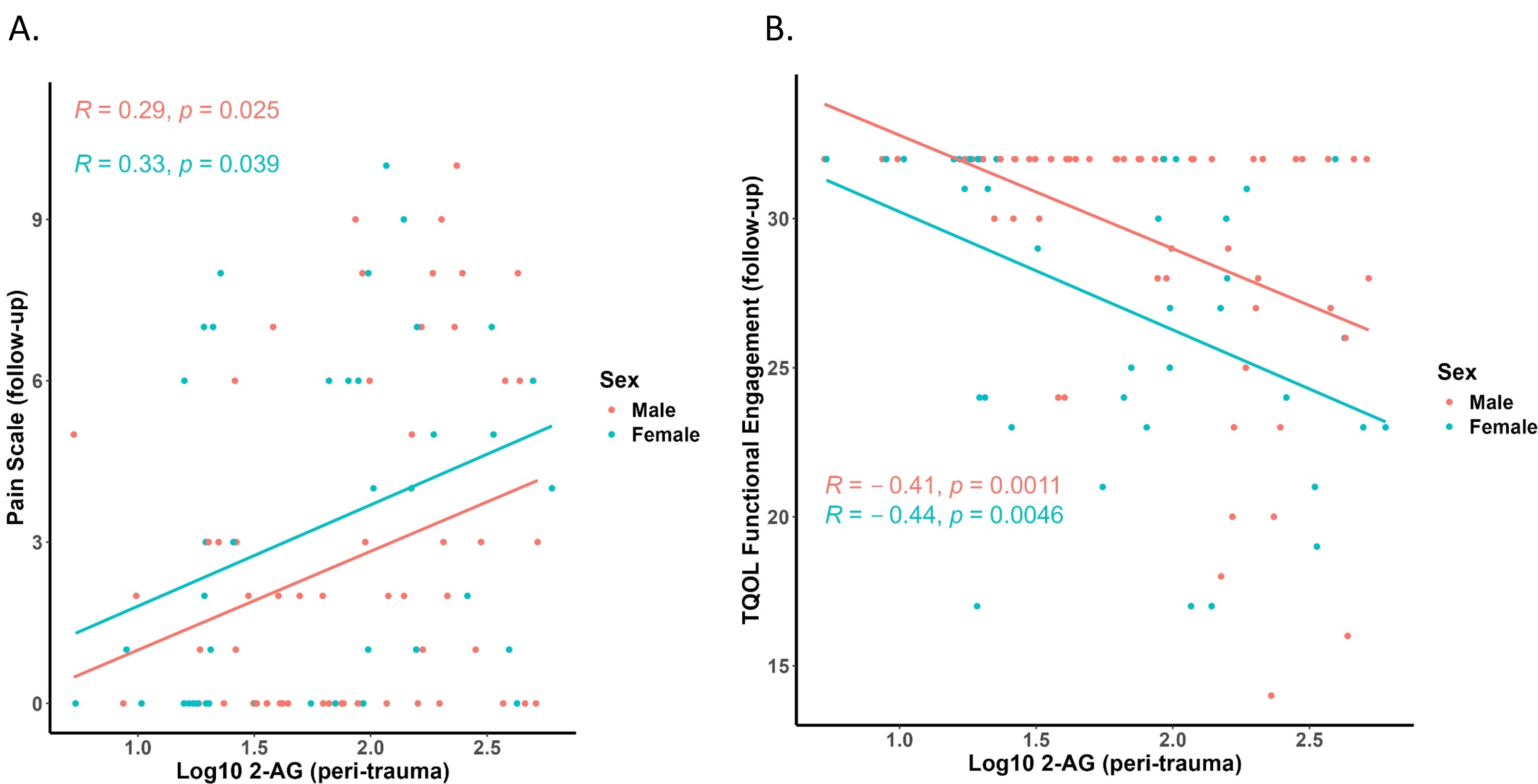


**Supplemental Figure 3.** Pain severity on a 10 point scale (A) and functional engagement subscores from the Trauma Quality of Life Survey (TQOL) (B) were measured at follow-up (6-10 months after the injury) and serum endocannabinoid concentrations were measured in blood harvested within several days of the injury (peri-trauma). Pink dots and lines represent data from male participants (n = 60) while blue dots and lines represent data from female participants (n = 40). Pearson’s R values and associated p values are reported on each panel for each sex.


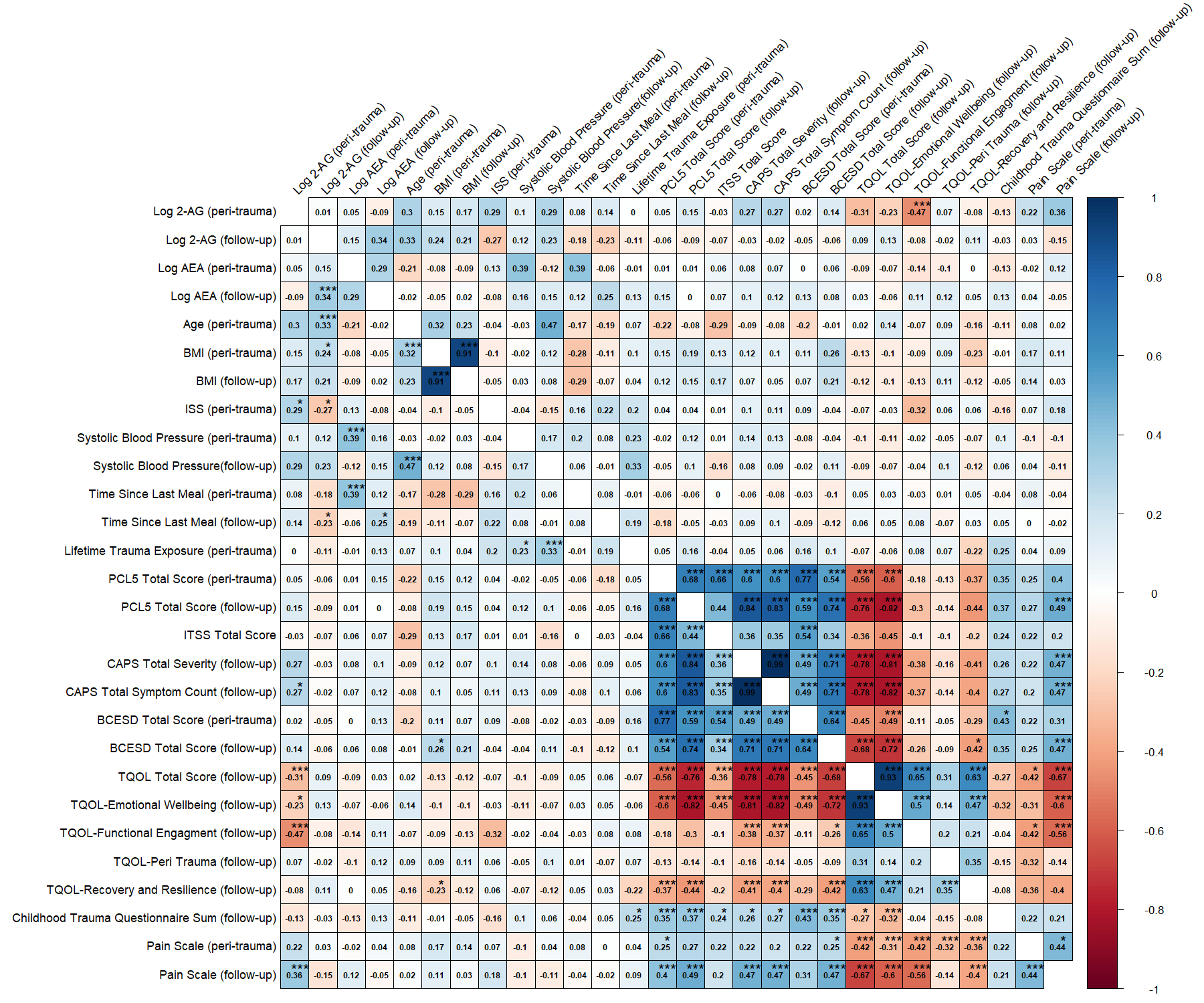


**Supplemental Figure 4.** Correlation matrix of serum endocannabinoid concentrations at both time points, Post Traumatic Stress Disorder symptoms, and selected demographic and clinical characteristics using person correlations. In the bottom triangle are uncorrected correlational values. 2-AG = 2-arachidonoylglycerol; AEA = N-arachidonoylethanolamine; peri-trauma = several days after injury; follow-up = 6–10 months following trauma; BMI = Body Mass Index; ISS = injury severity score; PCL-5 = PTSD Checklist for DSM-5; ITSS = Injury Trauma Survivor Screen; CAPS = Clinician-Administered PTSD Scale for DSM-5; BCESD = Brief Center for Epidemiological Studies-Depression; TQOL = Trauma Quality of Life Survey. Time from the last meal is hours from the last time eating to a blood. In the upper triangle include correlations that have been adjusted using Holm correction, associated p values are reported on each correlation: * p<0.05; ** p<0.01; *** p<0.001.

Supplemental Table 1. Comparison of participant characteristics with and without PTSD at follow-up

|  | No PTSD Dx (N=67) | Yes PTSD Dx (N=24) | T-Test or  Chi-Squared Test | Holm Adjusted p-value |
| --- | --- | --- | --- | --- |
| **Age (years)** |  |  | t = 2.19 * | n.s. |
| Mean (SD) | 39.1 (15.4) | 33.1 (10.0) |  |  |
| Median [Min, Max] | 36.7 [18.2, 76.5] | 30.4 [20.4, 57.4] |  |  |
| **Sex** |  |  | n.s. | n.s. |
| Male | 42 (62.7%) | 13 (54.2%) |  |  |
| **Racial Group** |  |  | χ ^2^= 5.40 | n.s. |
| Historically Racially/Ethnically Marginalized | 51 (76.1%) | 24 (100%) |  |  |
| **Body Mass Index (peri-trauma)** |  |  | n.s. | n.s. |
| Mean (SD) | 31.2 (7.19) | 33.6 (13.0) |  |  |
| Median [Min, Max] | 30.6 [16.8, 56.0] | 28.8 [19.7, 68.5] |  |  |
| Missing | 2 (3.0%) | 0 (0%) |  |  |
| **Body Mass Index (follow-up)** |  |  | n.s. | n.s. |
| Mean (SD) | 30.1 (8.64) | 31.4 (14.4) |  |  |
| Median [Min, Max] | 29.4 [0, 57.7] | 29.1 [0, 69.4] |  |  |
| Missing | 4 (6.0%) | 0 (0%) |  |  |
| **Mechanism of Injury** |  |  | χ ^2^= 7.85 | p = 0.02 |
| Assaultive | 14 (20.9%) | 13 (54.2 %) |  |  |
| Non-Assaultive | 53 (79.1%) | 11 (45.8%) |  |  |
| **Injury Severity Score** |  |  | n.s. | n.s. |
| Mean (SD) | 12.6 (8.95) | 14.9 (11.4) |  |  |
| Median [Min, Max] | 9.50 [1.00, 43.0] | 10.0 [4.00, 43.0] |  |  |
| Missing | 13 (19.4%) | 6 (25.0%) |  |  |
| **Life Events Checklist - Total Events (peri-trauma)** |  |  | n.s. | n.s. |
| Mean (SD) | 9.91 (7.05) | 10.7 (5.35) |  |  |
| Median [Min, Max] | 9.00 [0, 35.0] | 9.00 [2.00, 24.0] |  |  |
| **Life Events Checklist - Total Events (follow-up)** |  |  | n.s. | n.s. |
| Mean (SD) | 2.40 (4.37) | 3.33 (2.91) |  |  |
| Median [Min, Max] | 1.00 [0, 24.0] | 3.00 [0, 12.0] |  |  |
| **CESD-R Total Score (follow-up)** |  |  | t = -7.47 *** | p < .001*** |
| Mean (SD) | 8.07 (6.01) | 18.5 (5.82) |  |  |
| Median [Min, Max] | 6.00 [0, 25.0] | 18.5 [3.00, 27.0] |  |  |
| **CAPS5 Total Severity Mean Impute (follow-up)** |  |  | t = -14.24 *** | p < .001*** |
| Mean (SD) | 8.07 (7.12) | 35.6 (8.46) |  |  |
| Median [Min, Max] | 6.00 [0, 28.0] | 35.0 [20.0, 52.0] |  |  |
| **THC (Marijuana) Test (peri-trauma)** |  |  | n.s. | n.s. |
| Negative | 45 (67.2%) | 11 (45.8%) |  |  |
| Positive | 19 (28.4%) | 10 (41.7%) |  |  |
| Missing/Did Not Assess | 3 (4.5%) | 3 (12.5%) |  |  |
| **THC (Marijuana) Test (follow-up)** |  |  | n.s. | n.s. |
| Negative | 40 (59.7%) | 12 (50.0%) |  |  |
| Positive | 25 (37.3%) | 12 (50.0%) |  |  |
| Missing/Did not Assess | 2 (3.0%) | 0 (0%) |  |  |

Supplemental Table 2 Hierarchical Regression Results: Serum endocannabinoid concentrations at peri-trauma period and follow-up and CESD-R at follow-up

| **Predictor** | **Step 1** | **Step 2** | **Step 3** | **Step 1** | **Step 2** | **Step 3** |
| --- | --- | --- | --- | --- | --- | --- |
| Serum AEA – Peri-trauma | 0.089 | 0.182 | **.177*** | 0.066 | 0.147 | 0.16 |
| Serum 2AG – Peri-trauma | 0.065 | 0.136 | 0.07 | 0.063 | 0.135 | 0.084 |
| Age |  | -0.056 | 0.013 |  | -0.063 | 0.005 |
| Sex (0 = Male) |  | 0.292 | 0.077 |  | 0.194 | 0.034 |
| Race (0 = historically racially/ethnically marginalized) |  | -0.48 | -0.454 |  | **-0.509*** | **-0.473*** |
| Current Psychiatric Medication (0 = No) |  | 0.327 | 0.431 |  | 0.374 | 0.421 |
| Current Psychotherapy (0 = No) |  | -0.254 | -0.373 |  | -0.204 | -0.305 |
| Previous Psychiatric History (0 = No) |  | 0.239 | 0.128 |  | 0.232 | 0.128 |
| DASS Depression - Peri-trauma |  | **.366***** | **.345***** |  | **0.416***** | **0.378***** |
| PCL-5 Total - Peri-trauma |  | 0.183 | 0.058 |  | 0.172 | 0.045 |
| Days since injury – Peri-trauma |  | -0.006 | 0.0001 |  | -0.05 | -0.028 |
| Hours since food intake – Peri-trauma |  | -0.12 | -0.11 |  | -0.088 | -0.09 |
| THC urine tox screen - Peri-trauma (0 = negative) |  | -0.447 | -0.39 |  | **-0.541**** | **-0.447*** |
| LEC Weighted total – Peri-trauma |  | 0.034 | 0.031 |  | -0.002 | 0.003 |
| CAPS distress – Follow-up |  |  | **.423***** |  |  | **0.389***** |
| Childhood Trauma Questionnaire Sum – Follow Up |  |  |  |  |  | 0.013 |
| Constant | 0 | -0.034 | **-.512***** | -0.017 | 0.008 | **-0.445***** |
| Observations | 90 | 90 | 90 | 89 | 89 | 89 |
| R2 | 0.012 | 0.535 | 0.657 | 0.008 | 0.555 | 0.662 |
| Adjusted R2 | -0.011 | 0.448 | 0.587 | -0.015 | 0.471 | 0.586 |
| Residual Std. Error | 1.005 | 0.743 | 0.643 | 0.998 | 0.72 | 0.637 |
|  | (df = 86) | (df = 75) | (df = 74) | (df = 86) | (df = 74) | (df = 72) |
| F Statistic | 0.533 | **6.158***** | **9.434***** | 0.366 | **6.604**** | **8.797**** |
|  | (df = 2; 87) | **(df = 14; 75)** | **(df = 15; 74)** | (df = 2; 86) | **(df = 14; 74)** | **(df = 16; 72)** |

CESD-R = Center for Epidemiologic Studies of Depression Scale-Revised; AEA = *N*-arachidonoylethanolamine; 2-AG = 2-arachidonoylglycerol; DASS = Depression Anxiety and Stress Scale; PCL-5 = PTSD Checklist for DSM-5; THC = Tetrahydrocannabinol; LEC = Life Events Checklist for the Diagnostic and Statistical Manual of Mental Disorders; CAPS distress = responses to the question “overall, in the past month how much have you been bothered by these (PTSD Symptoms) you’ve told me about”. All values shown are unstandardized coefficients unless otherwise stated. * *p* < .05; ***p* < .025, ****p* <.01. Note the relationship between AEA and depression scores in Step 3 of the model without accounting for childhood trauma, the relationships between race and THC with depression scores in Step 2 and Step 3 of the model including childhood trauma, and the constant of Step 3 of the model including childhood trauma, did not remain significant following Holm’s Adjustment.

Supplemental Table 3: Hierarchical Regression Results: Peri-trauma serum endocannabinoid concentrations and CAPS-5 severity, Pain scores and Functional Engagement TQOL at follow-up with Childhood Trauma as a covariate

|  | **CAPS-5 Severity** | | **Pain Score** | | **Functional Engagement TQOL** | |
| --- | --- | --- | --- | --- | --- | --- |
| **Predictor** | **Step 1** | **Step 2** | **Step 1** | **Step 2** | **Step 1** | **Step 2** |
| Serum AEA – Peri-trauma | 0.108 | 0.157 | 0.483 | 0.294 | -0.15 | -0.198 |
| Serum 2-AG – Peri-trauma | 0.196 | **0.255**** | **1.043**** | **0.971**** | **-0.366***** | **-0.400***** |
| Age – Peri-trauma |  | -0.006 |  | 0.071 |  | -0.08 |
| Sex (0 = Male) |  | 0.134 |  | 0.425 |  | **-0.485*** |
| Race (0 = historically racially/ethnically marginalized) |  | -0.226 |  | -1.769 |  | 0.436 |
| Current Psychiatric Medication (0 = No) |  | -0.143 |  | 1.369 |  | -0.356 |
| Current Psychotherapy (0 = No) |  | 0.07 |  | -1.309 |  | 0.595 |
| Previous Psychiatric History (0 = No) |  | 0.374 |  | -1.199 |  | -0.09 |
| DASS Depression - Peri-trauma |  | -0.048 |  | -0.108 |  | 0.173 |
| PCL-5 Total - Peri-trauma |  | **0.562***** |  | 0.95 |  | -0.245 |
| Days since injury – Peri-trauma |  | 0.092 |  | 0.177 |  | -0.07 |
| Hours since food intake – Peri-trauma |  | -0.125 |  | -0.102 |  | 0.018 |
| Urine THC - Peri-trauma (0 = negative) |  | -0.042 |  | 0.394 |  | -0.005 |
| LEC Weighted total – Peri-trauma |  | -0.04 |  | 0.094 |  | 0.029 |
| Childhood Trauma Questionnaire Sum – Follow Up |  | 0.023 |  | 0.132 |  | -0.017 |
| Constant | -0.022 | -0.131 | **2.914**** | **3.208***** | 0 | 0.177 |
| Observations | 89 | 89 | 88 | 88 | 89 | 89 |
| R2 | 0.052 | 0.543 | 0.13 | 0.38 | 0.156 | 0.327 |
| Adjusted R2 | 0.03 | 0.449 | 0.109 | 0.251 | 0.136 | 0.189 |
| Residual Std. Error | 0.966 | 0.728 | 3.021 | 2.769 | 0.929 | 0.901 |
|  | (df = 86) | (df = 73) | (df = 85) | (df = 72) | (df = 86) | (df = 73) |
| F Statistic | 2.364 | **5.776**** | **6.330**** | **2.948**** | **7.948**** | **2.366*** |
|  | (df = 2; 86) | **(df = 15; 73)** | **(df = 2; 85)** | **(df = 15; 72)** | **(df = 2; 86)** | **(df = 15; 73)** |

TQOL= Total Quality of Life; AEA = *N*-Arachidonoylethanolamine; 2-AG = 2-Arachidonoylglycerol; DASS = Depression Anxiety and Stress Scale; PCL-5 = PTSD Checklist for DSM-5; THC = Tetrahydrocannabinol; LEC = Life Events Checklist for the Diagnostic and Statistical Manual of Mental Disorders; CAPS = Clinician-Administered PTSD Scale for DSM-5; All values shown are unstandardized coefficients unless otherwise stated. * *p* < .05; ***p* < .025, ****p* <.01. Note that the relationship between 2-AG and CAPS-5 severity scores did not remain significant following Holm’s adjustment. The Holm’s adjustment was not assessed for the pain and functional engagement models.
